# Late Visceral Herniation Secondary to Traumatic Left Hemidiaphragmatic Rupture After Blunt Trauma

**DOI:** 10.1155/cris/7160634

**Published:** 2026-05-19

**Authors:** J. Santiago Pabón-Castro, José Alejandro Díaz-Castillo, L. Fernando Vélez-Cuorvo, Johana C. Ramírez-Pérez, Valentina Velasco-Muñoz, Sara Prieto-Rodríguez

**Affiliations:** ^1^ Surgery Department, Sanitas University Foundation, Bogotá, Colombia, unisanitas.edu.co; ^2^ Medical School, Sanitas University Foundation, Bogotá, Colombia, unisanitas.edu.co; ^3^ Medical School, Pontificia Universidad Javeriana, Bogotá, Colombia, javeriana.edu.co

**Keywords:** blunt thoracoabdominal trauma, delayed diaphragmatic hernia, exploratory laparotomy, hemidiaphragmatic rupture, primary repair, stamm gastrostomy, thoracostomy, visceral herniation

## Abstract

Traumatic hemidiaphragmatic rupture (TDR) after blunt thoracoabdominal trauma is uncommon but potentially lethal if diagnosed late. The aim of this report is to highlight the delayed presentation of TDR and the diagnostic and therapeutic considerations in a resource‐limited setting. We present a case report of a 21‐year‐old man with obesity (BMI 30) suffered blunt trauma from a 2‐m fall. On initial evaluation, the chest X‐ray showed no hemidiaphragmatic elevation or pneumothorax, and he was discharged with recommendations. Fifteen days later, he was readmitted due to progressive dyspnea and desaturation. A Chest X‐ray and contrast‐enhanced computed tomography showed a left hemidiaphragmatic defect (46 × 70 mm) with herniation of the stomach, omentum, tail of the pancreas, and spleen. Exploratory laparotomy was performed, revealing a 10 cm × 7 cm defect with viable contents. Repair was achieved by primary closure with interrupted 0 polypropylene sutures, Stamm gastrostomy, and left thoracostomy. Postoperative evolution was satisfactory, and he was discharged after 12 days. This case is relevant because of its delayed presentation and the unusual inclusion of the pancreatic tail and spleen in the hernia sac, underscoring the need for a high index of suspicion and early imaging in closed thoracic trauma. TDR may present in a delayed fashion; early diagnosis by CT and timely surgical repair are essential to prevent severe complications, especially in resource‐limited contexts.

## 1. Introduction

Hemidiaphragmatic rupture due to blunt trauma is an unusual condition, with a reported incidence between 0.8% and 5.8% [1]. In our setting, it usually presents as a medical‐surgical emergency due to compression of pulmonary and mediastinal structures caused by herniation of abdominal viscera, with potential compromise of venous return. It generally affects the left hemidiaphragm in 50%–80% of cases [1], which is related to the anatomical protection afforded by the liver on the right side. In these cases, the most frequently herniated viscera include the stomach, spleen, omentum, pancreas, and splenic flexure of the colon [2]. Clinically, it may present with chest and/or abdominal pain, dyspnea, and decreased or absent breath sounds in the affected hemithorax.

Diagnosis requires a high index of suspicion, since this injury can be overlooked during the initial evaluation, contributing to an overall reported mortality of 13.7% [2]. Early repair of the defect is essential to prevent severe complications such as necrosis of the herniated contents, visceral perforation, and sepsis [2].

This article describes the clinical and radiologic diagnosis, as well as the surgical approach in a resource‐limited setting, through the presentation of a case of left hemidiaphragmatic rupture following blunt thoracoabdominal trauma.

## 2. Case Report

We present the case of a 21‐year‐old man with obesity (BMI 30) who sustained blunt thoracoabdominal trauma after a 2‐m fall. He reported left‐sided chest pain and mild dyspnea. On admission, he was hemodynamically stable, with oxygen saturation of 90% on room air. Chest radiography was interpreted as normal, without pneumothorax, hemothorax, or clear elevation of the left hemidiaphragm. As symptoms improved and there were no signs of intraabdominal injury, CT imaging was not performed, and the patient was discharged with return precautions.

On retrospective review, the initial chest radiograph showed no obvious signs of diaphragmatic disruption or visceral herniation, illustrating how these injuries may initially be radiographically occult.

**Figure 1:**
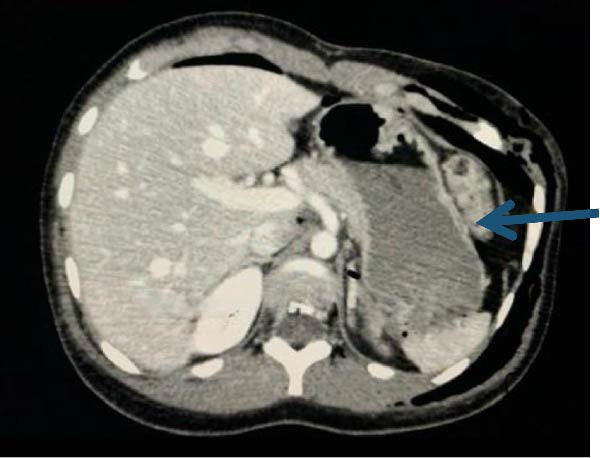
Axial computed tomography of the abdomen. The blue arrow shows the upward displacement of intestinal loops, suggesting hemidiaphragmatic rupture. *Source:* Image obtained during the study.

**Figure 2:**
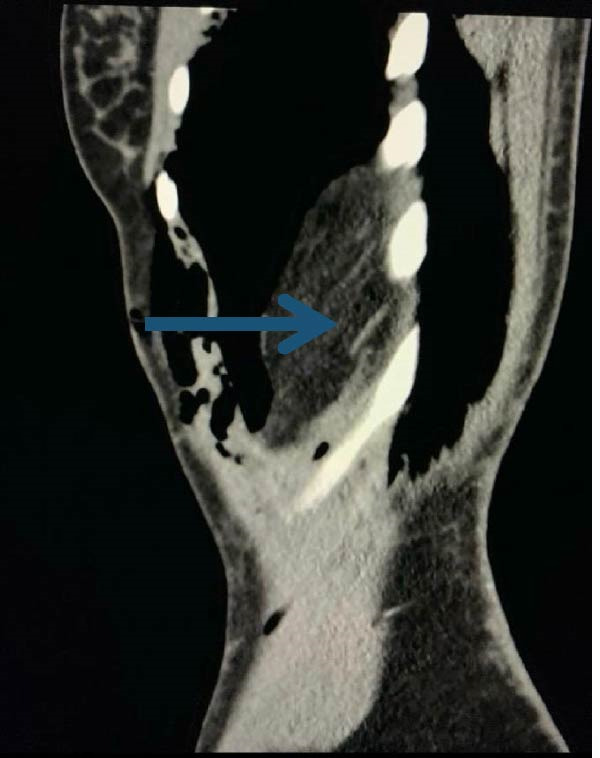
Sagittal computed tomography of the abdomen. The blue arrow shows hemidiaphragmatic disruption with herniation of abdominal viscera into the left hemithorax. *Source:* Image obtained during the study.

The patient returned 15 days later with progressive dyspnea, worsening left‐sided chest discomfort, left upper quadrant pain, abdominal distension, dysphagia, early satiety, and obstipation. Examination showed reduced breath sounds over the left hemithorax and mild abdominal distension without peritonitis. Repeat chest radiography demonstrated elevation of the left hemidiaphragm with apparent herniation of abdominal viscera and associated ipsilateral hemopneumothorax. Contrast‐enhanced CT confirmed a 46 mm × 70 mm defect in the left hemidiaphragm with herniation of the stomach, omentum, spleen, and pancreatic tail into the left hemithorax (Figures 1 and 2).

**Figure 3:**
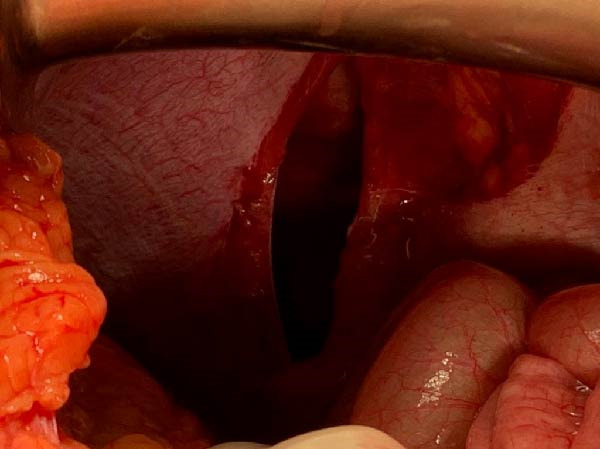
Open view of the diaphragm, showing a longitudinal rupture of ~10 cm. *Source:* Image obtained during the study.

**Figure 4:**
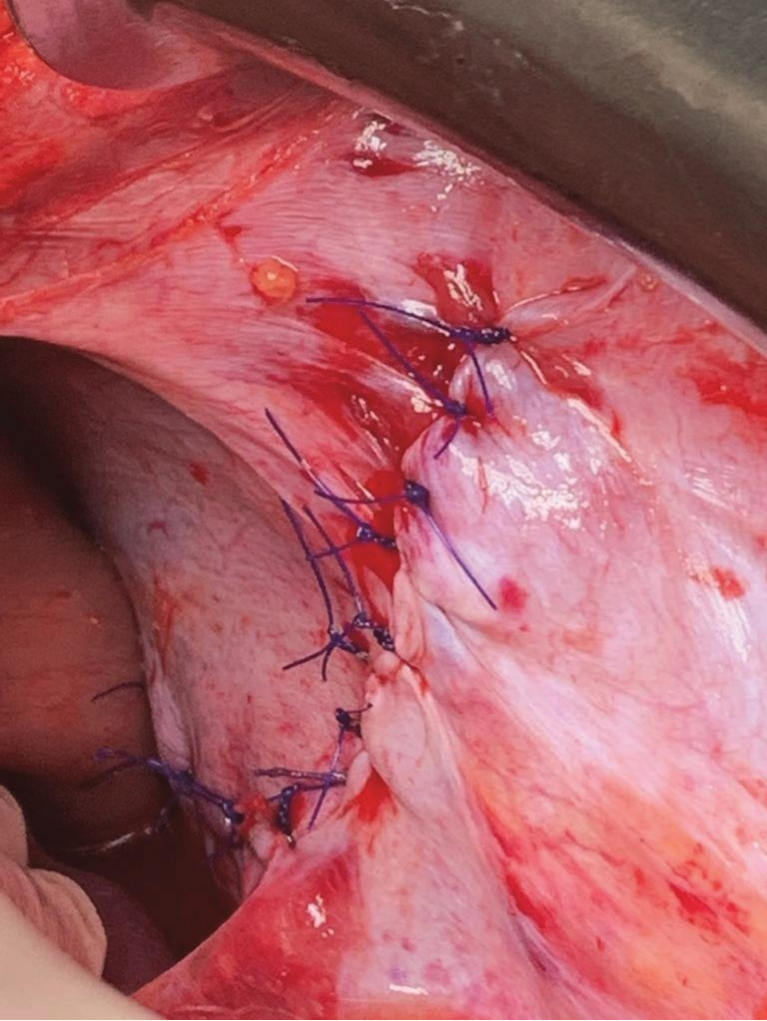
Open view of the diaphragm, showing primary closure with interrupted 0 polypropylene sutures. *Source:* Image obtained during the study.

Exploratory laparotomy was performed. After reduction of the herniated viscera, the stomach, spleen, and pancreatic tail were found to be viable, with no ischemia or perforation (Figures 3 and 4). The diaphragmatic defect measured ~10 cm × 7 cm and was repaired primarily with interrupted 0 polypropylene sutures. Mesh was not used because it was unavailable, although primary closure was achieved without significant tension. A Stamm gastrostomy was performed for gastric decompression, and a left tube thoracostomy was placed for the associated hemopneumothorax.

Postoperatively, the patient was managed in the critical care unit with analgesia, respiratory physiotherapy, and early mobilization. Oral intake was started on postoperative Day 2, the thoracostomy tube was removed on Day 6, and the gastrostomy tube on day 10. He was discharged after a total postoperative stay of 12 days. At the 30‐day follow‐up, he remained asymptomatic, with no dyspnea, abdominal symptoms, or clinical signs of recurrence. Radiologic follow‐up and longer‐term follow‐up were not available because the relevant images could not be retrieved and the patient did not return.

## 3. Discussion

Traumatic hemidiaphragmatic hernia is an uncommon consequence of blunt thoracoabdominal trauma and may be missed during the initial evaluation because both symptoms and radiographic findings can be subtle or absent [3–5]. In the present case, the patient initially had mild respiratory symptoms and an unrevealing chest radiograph but returned 15 days later with progressive dyspnea, upper abdominal symptoms, and imaging evidence of multivisceral herniation into the left hemithorax. This delayed presentation is consistent with the latent phase described in the literature and illustrates how these injuries may become clinically apparent only after herniation progresses [6, 7].

Chest radiography is usually the first imaging study in trauma, but its diagnostic yield is limited [4, 8]. In our patient, the initial radiograph did not show clear signs of diaphragmatic disruption, whereas repeat radiography raised suspicion and contrast‐enhanced CT established the diagnosis by demonstrating diaphragmatic discontinuity and herniation of the stomach, omentum, spleen, and pancreatic tail. This correlation between the initial negative radiograph and the later CT‐confirmed rupture highlights the importance of maintaining a high index of suspicion and considering early CT in patients with persistent or progressive symptoms after blunt thoracoabdominal trauma [4, 9–15].

The operative findings in this case were also clinically relevant. After reduction of the herniated viscera, no ischemia or perforation was identified despite delayed presentation, allowing definitive repair by laparotomy. This open approach was preferred because it allowed direct reduction of the herniated organs, assessment of visceral viability, and management of the associated hemopneumothorax. A laparoscopic approach was not considered because of the delayed presentation with multivisceral herniation, the need for direct evaluation of organ viability, and the resource limitations of our institution.

Although mesh reinforcement may be considered for larger defects, it was not used in this case. In addition to limited local availability, mesh may not have been mandatory because this was a traumatic rupture rather than a congenital diaphragmatic hernia, and therefore did not necessarily imply an intrinsic weakness of the diaphragmatic tissue itself. After complete reduction of the herniated contents, primary closure was achieved without significant tension and remained stable intraoperatively. This should be taken into account when comparing our management with reports from higher‐complexity centers, where prosthetic reinforcement and minimally invasive approaches may be more readily available [16–18].

## 4. Conclusions

Late presentation of traumatic hemidiaphragmatic rupture (TDR) may include multiorgan herniation (including the pancreatic tail). A high index of suspicion and early use of contrast‐enhanced chest/abdominal CT are essential for diagnosis; timely surgical repair, adapted to available resources (primary repair versus prosthetic reinforcement), reduces the risk of severe complications.

## Author Contributions

Conceptualization: J. Santiago Pabón‐Castro, José Alejandro Díaz‐Castillo, L. Fernando Vélez‐Cuorvo, and Johana C. Ramírez‐Pérez. Methodology: Valentina Velasco‐Muñoz and Sara Prieto‐Rodríguez. Investigation: J. Santiago Pabón‐Castro, José Alejandro Díaz‐Castillo, L. Fernando Vélez‐Cuorvo, Johana C. Ramírez‐Pérez, Valentina Velasco‐Muñoz, and Sara Prieto‐Rodríguez. Writing – original draft preparation: J. Santiago Pabón‐Castro, Valentina Velasco‐Muñoz, and Sara Prieto‐Rodríguez. Writing – review and editing: J. Santiago Pabón‐Castro, José Alejandro Díaz‐Castillo, L. Fernando Vélez‐Cuorvo, Johana C. Ramírez‐Pérez, Valentina Velasco‐Muñoz, and Sara Prieto‐Rodríguez. Visualization: J. Santiago Pabón‐Castro. Supervision: José Alejandro Díaz‐Castillo L. Fernando Vélez‐Cuorvo, and Johana C. Ramírez‐Pérez.

## Funding

This research received no external funding.

## Disclosure

All authors have read and agreed to the published version of the manuscript. The authors have reviewed and edited the output and take full responsibility for the content of this publication.

## Consent

Informed consent was obtained from all subjects involved in the study. Written informed consent has been obtained from the patient to publish this paper.

## Conflicts of Interest

The authors declare no conflicts of interest.

## Data Availability

The data that support the findings of this study are available upon request from the corresponding author. The data are not publicly available due to privacy or ethical restrictions.
